# Radiobiological Evaluation of Combined Gamma Knife Radiosurgery and Hyperthermia for Pediatric Neuro-Oncology

**DOI:** 10.3390/cancers13133277

**Published:** 2021-06-30

**Authors:** Morteza Ghaderi Aram, Massimiliano Zanoli, Håkan Nordström, Iuliana Toma-Dasu, Klas Blomgren, Hana Dobšíček Trefná

**Affiliations:** 1 Department of Electrical Engineering, Chalmers University of Technology, 412 96 Gothenburg, Sweden; zanoli@chalmers.se (M.Z.); hanatre@chalmers.se (H.D.T.); 2 Elekta Instrument AB 113 57, 103 93 Stockholm, Sweden; hakan.nordstrom@elekta.com; 3 Department of Oncology-Pathology, Karolinska Institutet, 171 64 Stockholm, Sweden; iuliana.livia.dasu@ki.se; 4 Department of Physics, Stockholm University, 106 91 Stockholm, Sweden; 5 Department of Women’s and Children’s Health, Karolinska Institutet, 171 64 Stockholm, Sweden; klas.blomgren@ki.se; 6 Pediatric Oncology, Karolinska University Hospital, 171 64 Stockholm, Sweden

**Keywords:** stereotactic radiotherapy, hyperthermia, CNS tumors, medulloblastoma, biological modelling, LQ model

## Abstract

**Simple Summary:**

This study proposes a novel strategy in brain cancer management. Stereotactic radiosurgery delivered by the Gamma Knife was combined with hyperthermia. For the radiobiological modelling of this synergistic treatment modality, we used the linear-quadratic model with temperature-dependent parameters to assess the potential enhancement of the therapeutic outcome. The results indicate that focused intracranial heating can be used to boost the dose to the target. Alternatively, one can conclude that for the same therapeutic effect, hyperthermia can help to minimize the dose undesirably delivered to healthy tissues. This study is also the first to advocate a combination of stereotactic radiosurgery with focused heating and motivates the future development of hyperthermia systems for brain cancer treatment.

**Abstract:**

Combining radiotherapy (RT) with hyperthermia (HT) has been proven effective in the treatment of a wide range of tumours, but the combination of externally delivered, focused heat and stereotactic radiosurgery has never been investigated. We explore the potential of such treatment enhancement via radiobiological modelling, specifically via the linear-quadratic (LQ) model adapted to thermoradiotherapy through modulating the radiosensitivity of temperature-dependent parameters. We extend this well-established model by incorporating oxygenation effects. To illustrate the methodology, we present a clinically relevant application in pediatric oncology, which is novel in two ways. First, it deals with medulloblastoma, the most common malignant brain tumour in children, a type of brain tumour not previously reported in the literature of thermoradiotherapy studies. Second, it makes use of the Gamma Knife for the radiotherapy part, thereby being the first of its kind in this context. Quantitative metrics like the biologically effective dose (BED) and the tumour control probability (TCP) are used to assess the efficacy of the combined plan.

## 1. Introduction

Central nervous system (CNS) tumours constitute the second most common form of cancer in children [1]. Improved treatment protocols have increased survival rates to more than 80% [2]. However, the treatment may have multiple, debilitating side effects, so-called late effects or late complications. Radiotherapy (RT) is an essential treatment modality in the clinical management of brain tumours and vascular malformations, but it is also most prone to cause late complications. Long-term neurocognitive sequelae are often severe in paediatric patients, where 50–96% of the treated individuals display intellectual impairments [3,4]. Methods such as proton therapy and stereotactic treatments with photons are becoming increasingly popular, especially in children, because of their potential to deliver a dose limited to the target and thus reduce RT-related toxicity.

Hyperthermia (HT) is defined as an induced temperature increase in tumours to about 40–44 °C for typically 60 min. It has been demonstrated that hyperthermia (HT) improves local control for a wide range of tumours and can increase overall survival rates in patients treated with radiotherapy (RT) [5,6,7,8,9] or chemotherapy [10,11]. For instance, despite the technically challenging delivery of HT to the head and neck (H&N) region, HT offers a valuable treatment option for patients with tumours in this region [12]. A meta-analysis study of H&N carcinomas, recently published in [7], shows that the complete response rate (CR) in patients treated with combined radiotherapy and hyperthermia (RTHT) appears to be significantly better than that of the patients treated with RT alone (RT alone: CR = 39.6% vs. RTHT: CR = 62.5%). At the same time, acute and late-grade 3/4 toxicities have not been reported to be significantly different between two arms [7]. Although most of the patients treated with hyperthermia have been adults, encouraging clinical results also exist for children and adolescents [13]. The combination of chemotherapy and hyperthermia has been successfully applied in children with refractory or recurrent non-testicular malignant germ cell tumours. The long-term prognosis for patients with poor response or after the first relapse is similar to the prognosis for those receiving first-line treatment [14].

The clinical experiences with HT applied for brain cancer treatment are limited to high-grade glioblastomas (GBM). The interstitial HT combined with radiotherapy has been demonstrated to be a safe [15,16,17] and promising modality to improve the survival of the patients. In a two-arm study, Sneed et al. [18] have shown a two-year survival rate of 31% in the arm with HT versus 15% in the arm without HT. Despite the promising results in terms of an improved treatment outcome, homogeneous tumour temperatures were difficult to achieve with the interstitial applicators used [15,18]. As an adjuvant for RT, intratumoral injection of magnetic nanoparticles in magnetic hyperthermia therapy has also been shown to improve survival rate in GBM, compared to the therapeutic outcomes achieved with RT alone [19]. More recently, a localised increase of tumour temperature has been achieved through magnetic fluid HT [20], in which injected iron oxide nanoparticles are excited by an externally applied AC magnetic field. Again, the higher overall survival of patients in the arms including HT was observed [21]. Another technology with promising results in moderate heating in small animal tumours is the high-intensity focused ultrasound (HIFU) [22]. The present HIFU technology has not yet been proven feasible for the heating of large volumes typical for childhood brain tumours.

Our work is based on microwave phased array technology [23,24] that has been the most widely applied technology for the treatment of deep-seated tumours in clinical settings. However, this technology has not been applied intracranially before due to several challenges. The high perfusion rates in the brain quickly normalise the temperature and thus require the application of strong electromagnetic (EM) fields to achieve adequate tumour temperatures. Moreover, the presence of electrically highly conductive cerebrospinal fluid (CSF) leads to considerable absorption of EM radiation, thereby making CSF more susceptible to the emergence of treatment-limiting hot spots. However, recently published numerical studies [25,26,27,28,29] suggest that the intracranial heating with those innovative approaches is feasible. A particularly unique approach is a helmet-like configuration that utilises UWB antennas, allowing for better focusing with fewer antennas than standard annular-phased-array applicators [30]. Using a thermodynamic fluid model of CSF, the study demonstrated that this type of applicator is capable of obtaining an adequate temperature in large brain tumours without inducing unacceptable hot spots [31].

In current clinical practice, HT is typically delivered in combination with external beam radiation therapy (EBRT), which strongly relies on the fact that normal tissues are generally better at sublethal DNA damage repair than tumour tissues. Therefore, numerous dose fractionation schemes, in the order of 30 fractions delivered over a period of 5–10 weeks, have been exploited [32]. In comparison, stereotactic radiosurgery (SRS) aims to achieve a therapeutic effect by using a single or few fractions to deliver a highly conformal dose. Since its invention, the Leksell Gamma Knife^®^ (Elekta AB, Stockholm, Sweden) models have been used to sculpt the dose to conform to the target. The dose gradient outside the target is sharp, with little impact on surrounding healthy tissues and organs at risk. The SRS delivery is often limited to small target volumes, since its application for medium-sized and large tumours eradication would lead to an unfeasible increase in the treatment time.

In this study, we propose a novel strategy in brain cancer management, combining hyperthermia with SRS delivered by the Gamma Knife. The hypothesis is that focused microwave heating of a tumour will boost the radiotherapeutic effect or alternatively allow for a reduced radiation dose without compromising the treatment outcome. The potential treatment enhancement is investigated via radiobiological modelling, specifically via the linear-quadratic (LQ) model adapted to thermoradiotherapy through modulating the radiosensitivity of temperature-dependent parameters [33,34]. The model is further extended with an oxygen modification factor (OMF) that includes the effect of the local oxygen tension, $pO_{2}$ [35]. The model is applied to evaluate the expected increase of the therapeutic window when hyperthermia is added to SRS. The combined effect is assessed on a medium-sized paediatric brain tumour. The methodology proposed in this work can be considered as a framework for the evaluation of the combined effect of thermal therapy and radiosurgery.

## 2. Materials and Methods

### 2.1. Patient Model

**Figure 1:**
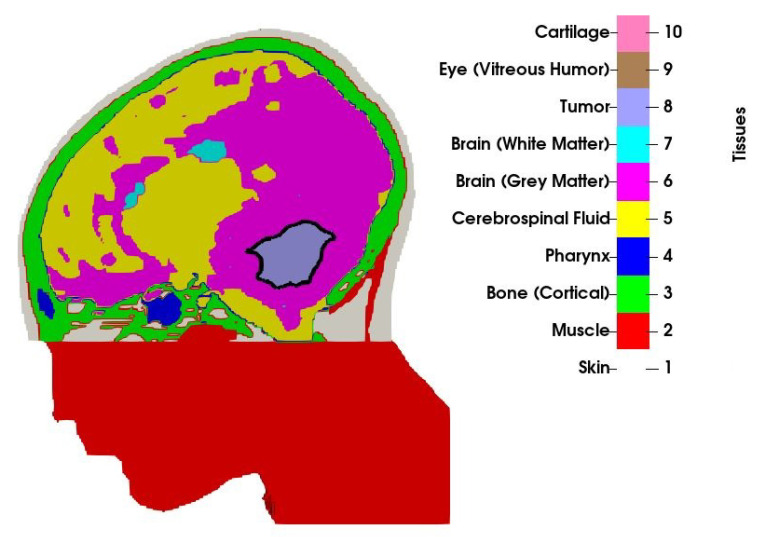
Cross-section of the patient model along with the tissue indices. Border of the tumour is shown with the solid black line.

An MRI scan with a 1×1×1 mm resolution was obtained from a 13-year old boy with medulloblastoma. The scan was manually segmented by a clinician into 10 tissue types, as visualized in Figure 1. Only a part of the head was segmented, and the section outside the treatment volume, that is, the part of the head below the tumour that was not covered by the applicator was modelled as muscle. Observe that the volume of the original tumour, as studied in [31], has been reduced to 34mL by means of morphological operations (erosion). The volume no longer occupied by the tumour has been filled with a mirrored copy of the opposite healthy brain hemisphere to preserve the anatomical correctness. In the context of the current proof-of-concept study, this may be considered a valid model for various residual brain tumours, such as medulloblastomas or ependymomas.

Electromagnetic and thermal simulations are performed using tissue parameters from the IT’IS database [36] adjusted for hyperthermic conditions: muscle perfusion is increased by a factor of 4 due to the systemic response to heat [37], while the thermal conductivity of the cerebrospinal fluid is increased by a factor of 10 to emulate the convective transport of heat [31]. Tumour properties are obtained as an average of grey and white matter, and its blood perfusion is decreased by a factor of 0.7 to account for the chaotic vasculature [38]. Due to the frequency dependence of the dielectric properties and utilisation of multiple frequencies in the treatment planning phase, the properties are not listed here. Nevertheless, the tissue properties listed in [31] can be considered as an example for 450 MHz.

### 2.2. Hyperthermia Treatment Planning with Novel Applicator

The hyperthermia applicator consists of eight self-grounded bow-tie antennas immersed into a separate water bolus [39] and arranged in a helmet-shaped array. A water bolus is inserted between the antennas and the patient for skin-cooling and impedance-matching. Four antennas operate across the frequency band of 400∼800 MHz, while the others are upscaled to operate at lower frequencies of 300∼600 MHz. The applicator is thus designed for multi-frequency treatments, and the set of operating frequencies considered for this study is 300,400,500,600,700, and 800MHz. Each antenna is excited by a periodic signal resulting from the superposition of all the individual frequencies. Each frequency component is independently steered in phase and amplitude for each antenna.

**Figure 2:**
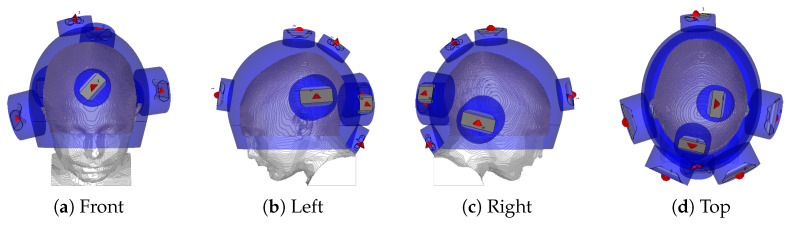
Helmet applicator optimized for the thermal treatment of the tumour considered in this study. The red cones indicate the feed point and polarization direction of each antenna. The blue shade indicates water.

The antenna arrangement within the applicator is obtained via a global optimization procedure based on a specific absorption rate (SAR) that, conjointly for each antenna, determines the location and polarization angle to minimize the hot-spot to target quotient (HTQ) [40]. The procedure has been introduced in [41] and subsequently extended for use with the non-linear cost function HTQ and for the multi-frequency ultra-wideband (UWB) range adopted in this study. The steering parameters, that is, the phase and amplitude of each antenna at each operating frequency, are obtained via particle swarm optimization [42], using the HTQ as the cost function. The final antenna arrangement is visualized in Figure 2. The temperature distribution is then obtained by scaling the power deposition until the threshold for thermal damage in healthy brain temperatures (42°C) [43] is reached. The temperature of the water bolus is set to 10°C. The quality and feasibility of the treatment plan are evaluated in terms of the indexed temperatures $T_{10}$, $T_{50}$ and $T_{90}$, which represent the temperatures achieved in 10, 50, and 90% of the target volume, respectively.

### 2.3. Stereotactic Radiosurgery Treatment Planning

The Gamma Knife models, Leksell Gamma Knife Icon^®^ Icon^TM^ and Leksell Gamma Knife^®^ Perfexion^TM^ (Icon, Perfexion), deliver dose from 192 Cobalt-60 sources that are collimated into narrow beams by a large tungsten body, see, for example, [44]. The beams converge in a small volume, called the isocenter, about 40 cm from the sources. The sources are housed within eight sectors with 24 sources in each sector. The sectors can slide over the collimator body, which in total has 576 collimator channels, in four different positions to produce beams of sizes of 4, 8 and 16 mm (The sizes refer to the cross-sectional diameters of single beams at the isocenter). The fourth position is beam-off when only a negligible amount of radiation leaks through the tungsten body. For each isocenter there are 65,535 different beam size combinations, colloquially called “shots”. By shifting the position of the patient, a dose is delivered to several isocenters. The total dose can thus be sculpted to conform to the target with a sharp dose gradient outside the target, leading to a small dose to healthy tissue and organs at risk.

To create a dose plan, patient images are imported to the treatment-planning software Leksell GammaPlan^®^ (LGP). In LGP, the target and organs at risks (OAR) are outlined, and the shots are placed in the target and weighted relative to each other to create an adequate dose distribution. The dose plan to the tumour described above was created by the new optimization tool, Leksell Gamma Knife^®^ Lightning. By specifying the prescription dose to the target, max dose constraints on OARs, and optimization weights, a plan with a reasonable trade-off between quality and beam-on-time (BOT) is determined.

For this particular case no specific OAR is outlined, instead bringing down the dose to the tissue surrounding the target is promoted. Furthermore, achieving a plan with good-quality metrics was considered to be the most important objective, and hence less emphasis was put on bringing down the BOT. In Table 1 the quality metrics and beam-on-time are given for this plan. For definitions of radiosurgical metrics, see [45]. Note that achieving high target dose homogeneity is seldom an objective in Gamma Knife surgery. On the contrary, for most plans, the prescription dose at the periphery of the target corresponds to 40–60% of the max dose in the target. This is to ensure a sharp dose gradient at the target periphery leading to a rapid fall-off of the dose. In this particular case, the relative isodose of 60% was chosen by the optimizer.

### 2.4. Radiosensitivity Modelling

The overall cell survival of the combined therapy can be described by a generalised LQ model that includes both direct cytotoxicity and a radiosensitising effect of hyperthermia. The cell survival is expressed as a function of temperature (*T*) and radiation dose (*D*), as well as the time interval between the two therapies:

(1) $$SF\left(D,T,t_{int}\right)=SF_{HT}\left(T\right)\times SF_{RT}\left(D,T,t_{int}\right),$$

where $SF_{HT}$ is the term referring to cell-killing due to direct hyperthermic cytotoxicity, while $SF_{RT}$ accounts for cell-killing due to radiation. $t_{int}$ is the time interval between the end of radiotherapy and the start of hyperthermia treatment in the range of [0–4] h.

$SF_{HT}$ can be modeled using the Arrhenius relationship [46]:

(2) $$SF_{HT}\left(T\right)=exp\left[-K\left(T\right)\times t_{H}\right],$$

where $t_{H}$ is the heating time (in this case 1 h) and *K* is the reaction rate as a function of temperature *T* (°C), given by [33]:

(3) $$K\left(T\right)=2.05\times 10^{10}\times \left(T+273.15\right)\times exp(\frac{\Delta S}{2}-\frac{\Delta H}{2(T+273.15)}),$$

where ΔH (cal/mol) is the inactivation energy of the critical rate-limiting molecules which are responsible for cell death, and ΔS (cal/°C/mol) is the entropy of inactivation.

For the $SF_{RT}$ part, the extended LQ model, which considers the radiosensitising effect of HT, is used to describe the cell killing due to radiotherapy, according to [33]

(4) $$SF_{RT}\left(D,T,t_{int}\right)=exp(-\alpha \left(T,t_{int}\right)\times D-G\times \beta \left(T,t_{int}\right)\times D^{2}),$$

with *G* as the protraction factor as defined in Section 2.5 and

(5) $$\alpha \left(T,t_{int}\right)=\alpha {}_{37}\times exp\left(\frac{T-37}{41-37}\times ln\left(\frac{\alpha {}_{41}}{\alpha {}_{37}}\right)\times exp(-\mu .|t_{int}\left|\right)\right),$$

(6) $$\beta \left(T,t_{int}\right)=\beta {}_{37}\times exp\left(\frac{T-37}{41-37}\times ln\left(\frac{\beta {}_{41}}{\beta {}_{37}}\right)\times exp(-\mu \times |t_{int}\left|\right)\right),$$

where μ (h${}^{-1}$) is the rate at which the radiosensitizing effect of hyperthermia disappears, $\alpha {}_{37}=\alpha \left(37,0\right)$, $\alpha {}_{41}=\alpha \left(41,0\right)$, $\beta {}_{37}=\beta \left(37,0\right)$, and $\beta {}_{41}=\beta \left(41,0\right)$.

In this study, we applied parameters for a generic head and neck (H&N) tumour [47], as reported in Table 2. The parameters for healthy tissues are same as tumour parameters with two distinctions: (a) at normothermic temperatures $\alpha {}_{37}/\beta {}_{37}=3$ Gy, a ratio which is well-established, (b) μ=1 h${}^{-1}$ as the radiosensitising effect tends to disappear faster in normal tissue than in tumorous tissue [48]. The alpha and beta ratios at elevated temperatures, that is, $\alpha {}_{41}/\alpha {}_{37}$ and $\beta {}_{41}/\beta {}_{37}$, are kept the same as for the tumour model since we could not find any experimental data for them in the literature.

#### The Oxygen Effect

The cell survival model, as described in the preceding section, considers that all the cells in the population are well-supplied with oxygen, hence a fully oxic cell population. To assess the effect of molecular oxygen on the irradiated tissue, the oxygen enhancement ratio (OER) is defined as the ratio of radiation dose in hypoxia to that of in well-oxygenated conditions. Based on OER, oxygen modification factors (OMF) being dependent on both the local oxygen tension ($pO_{2}$) and the duration of hypoxia ($t_{hyp}$) can be incorporated in the linear and quadratic parameters of the model, that is, α and β, as follows [49]:

(7) $$\alpha {}_{hyp}=\frac{\alpha (T,t_{int})}{OMF(pO_{2},t_{hyp})}$$

(8) $$\beta {}_{hyp}=\frac{\beta (T,t_{int})}{OMF^{2}\left(pO_{2},t_{hyp}\right)}.$$

In this study, it was assumed that the cell oxygenation is not changing during the course of the treatment. Hence, only the $pO_{2}$ effect has been taken into account and the time factor is omitted by adopting the following OMF proposed by Alper and Howard-Flanders [35]:

(9) $$OMF\left(pO_{2}\right)=OER_{max}\frac{k+pO_{2}\left(r\right)}{k+OER_{max}.pO_{2}\left(r\right)},$$

where *k* is a reaction constant of 2.5–3 mmHg [50,51], while $OER_{max}$ is the maximum protection achieved in the absence of oxygen which is considered to be 3 here.

**Figure 3:**
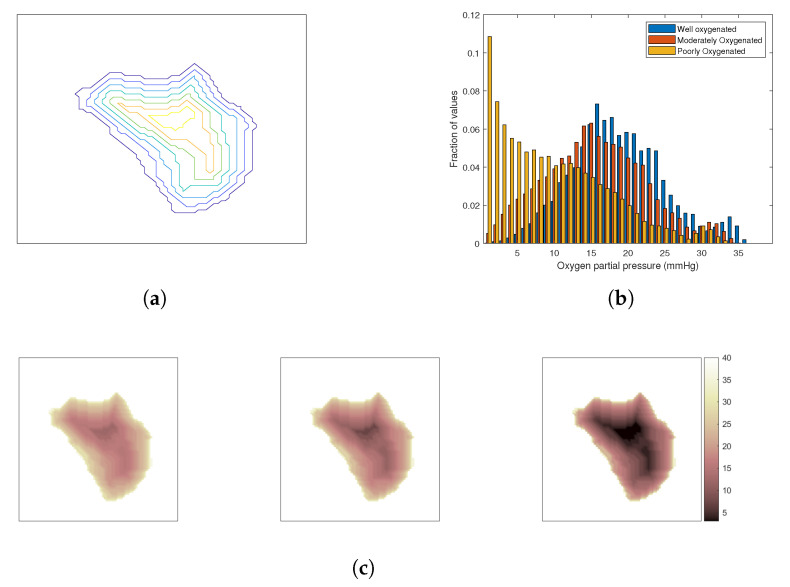
Modelling of the oxygenation level in the tumour. (**a**) A transverse cut of the iso-contour from the surface of the tumour. (**b**) Clinically representative histograms of $pO_{2}$ for a generic tumour under three different hypoxic conditions. (**c**) Mapped $pO_{2}$ distribution for those that are well-, moderately-, and poorly-oxygenated, respectively, from left to right.

The oxygenation of the tumour depends on its vasculature, which is chaotic and irregular [52]. Tumours are often characterized by a poorly oxygenated core due to the lack of blood vessels reaching the deeper layers that are surrounded by regions of progressively increased oxygenation towards the tumour periphery. To investigate the impact of oxygenation effect on the outcome of combined HT and SRS treatment, three cases have been considered: a well-oxygenated tumour, a moderately oxygenated tumour, and a poorly oxygenated tumour. The tumour was segmented into several iso-distance layers from the periphery inwards as shown in Figure 3a and a degree of oxygenation was allocated to each layer from a set of three distributions of oxygen partial pressure in tissue [53], Figure 3b. Figure 3c visualizes the resulting distributions of oxygen partial pressure in the considered models.

### 2.5. Evaluation of Effect of the Combined Treatment

In order to quantify the impact of combined treatment, we consider two metrics: the equivalent normalized total dose (EQD) and biological effective dose (BED). Although these two metrics are related to each other, a clear distinction between them is necessary. Supposing an SRS schedule with *n* fractions of equal size, complete repair between fractions, and negligible repair during the fractions, the protraction factor is given by G=1/n. The EQD can then be calculated by solving the following equation

(10) $$SF\left(EQD\right)=SF\left(D,T,t_{int}\right),$$

which results in a second-order equation in terms of EQD and yields the following formula:

(11) $$EQD=\frac{-\alpha {}_{37}+\sqrt{\alpha {}_{37}^{2}+4G\beta {}_{37}\times \left[\alpha \left(T,t_{int}\right)\times D+G\times \beta \left(T,t_{int}\right)\times D^{2}+K\left(T\right)\times t_{H}\right]}}{2G\beta {}_{37}},$$

where $\alpha (T,t_{int})$ and $\beta (T,t_{int})$ are given in Equations (7) and (8), respectively. The BED given for the fractionated plan with $d_{frac}$ as the dose per fraction is then calculated using BED=EQD×RE where RE stands for relative effectiveness given by $RE=1+\frac{d_{frac}}{\alpha /\beta }$ [47].

In our analysis, we further considered tumour control probability (TCP), an additional metric that estimates the probability that a tumour will be eradicated or controlled by the thermoradiotherapy. In particular, the TCP describes the probability with which cancer cells will be killed by a given radiation treatment dose *D* and can be described by a Poisson-based function [54]:

(12) $$\prod_{i=1}^{N_{T}}{\left(1-SF_{i}\right)}^{n_{i}}\approx \prod_{i=1}^{N_{T}}exp\left(-n_{i}.SF_{i}\right),$$

where $N_{T}$ is the total number of voxels in tumour and $n_{i}=n$ is the number of clonogenic cells.

The potential effect of the combined treatment on the normal brain tissue is evaluated by assessing the clinically relevant parameter $V_{10}$ volume. The $V_{10}$ volume is defined as the volume of the brain tissue outside the target that receives a BED $\geq 10\;(1+\frac{10}{{\left(\alpha /\beta \right)}_{Healthy}})$ Gy. Hence, in the forthcoming analysis, $V_{10}$ is defined as a region with BED≥43 Gy, and its estimation is limited to the resolution of the voxel model, that is, 1 (mm${}^{3}$).

## 3. Results

**Figure 4:**
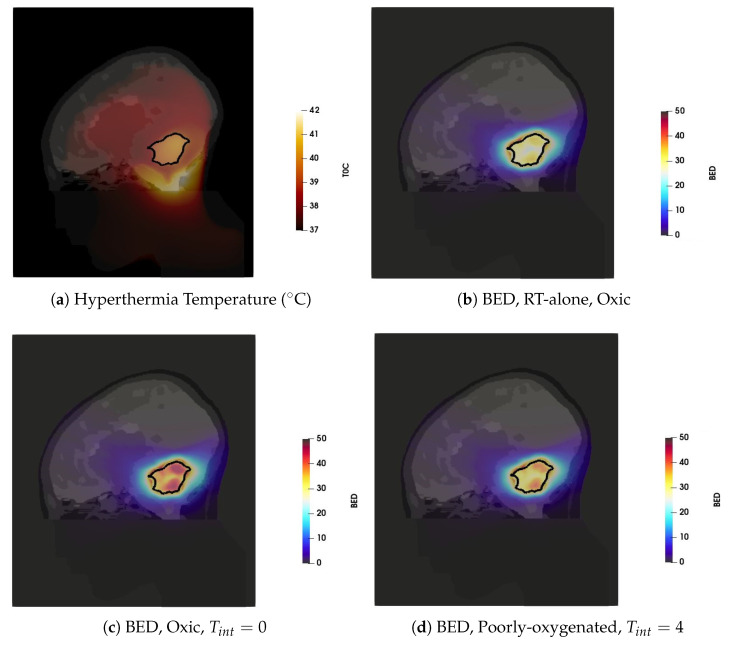
The thermal and BED distributions in the sagittal plane (**a**) Temperature distribution. (**b**) BED for RT plan with the total dose of 15 Gy delivered in five-fractions (**c**) BED of the combined plan for the well-oxygenated tumour and sequential administration with $T_{int}=0$ (**d**) BED of the combined plan for the poorly oxygenated tumour and HT administration 4 h after RT.

The hyperthermia treatment plan was obtained by using the SAR optimization procedures, which resulted in HTQ values of 1.5 and excellent tumour coverage ${\mathrm{TC}}_{25}=98\%$. The resulting steady-state temperature distribution, visualized in Figure 4a, was then obtained by scaling the power deposition with hard constrains for normal tissue temperature of 42 °C. The temperature distribution is visualized in the sagittal plane of the patient model, with the tumour delineated by a solid black line. Note that only temperatures above 37 °C are shown for better visualization. Temperatures below 37 °C are caused by the surface cooling water bolus and do not have any impact on the analysis of the combined RT + HT effect. The main hot-spot (i.e., the tissue temperature 42 °C) is located in the pocket of cerebrospinal fluid caudal to the target volume. The achieved $T_{90}=39.0\;{}^{\circ}C$, $T_{50}=39.8\;{}^{\circ}C$ and $T_{10}=40.4\;{}^{\circ}C$ indicate adequate tumour coverage by the thermal dose.

The Gamma Knife treatment plan with the prescribed dose of 15 Gy resulted in 99.4% coverage, as reported in Table 1, and BED of 37.5 Gy when delivered in a single fraction. Given the size of the target, this treatment plan resulted in an unacceptably high $V_{10}$ of 43 cm${}^{3}$. In order to mitigate this issue, a five-fraction scheme is considered in the analysis. Observe that we used a simple, uncompensated scheme where the original treatment dose is maintained despite the fractionation. The compensation is considered later in this section. In all cases of the fractionated scheme, we assume that HT is delivered after each RT fraction, provided that the time between the RT fractions is long enough to allow for sublethal damage reparation and for prevention of development of the thermotolerance [55,56]. The BED distribution corresponding to the five-fraction scheme is visualized in Figure 4b. One can observe a sharp dose gradient around GTV that is characteristic of SRS treatments.

**Figure 5:**
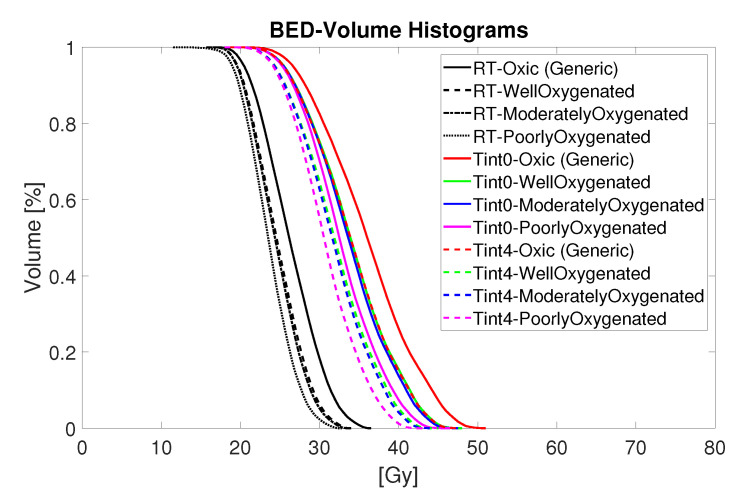
BED-Volume histograms for thermoradiotherapy plan with a total dose of 15 Gy given in five fractions.

The boosting effect of adjuvant hyperthermia on the BED is illustrated on two cases: heat applied to fully oxic tumour directly after irradiation (Figure 4c) and heat applied to poorly oxygenated tumour four hours after radiation (Figure 4d). These cases represent the extreme values of the enhanced BED achieved for all considered cases shown in Figure 5. In both cases, the thermoradiotherapy plan resulted in a substantially higher BED to the GTV than the radiosurgery-only plan.

Figure 5 summarizes in detail the estimated biological effective radiation dose of the combined thermoradiotherapy treatment plan (RT + HT) achieved for different oxygenation conditions and sequential administration. The black lines represent the results for radiation only, while the coloured lines represent the combined treatment administered with time interval 0 (solid coloured lines) and 4 h after irradiation (dashed coloured lines). Although the results suggest a noticeable increase in the BED values for all levels of oxygenation, the administration of HT directly after RT, that is, $T_{int}=0$, yields a bigger boost for each oxygenation level. Furthermore, the highest BED is observed for the oxic population, followed by the estimated BED for well-, moderately-, and poorly-oxygenated scenarios.

**Figure 6:**
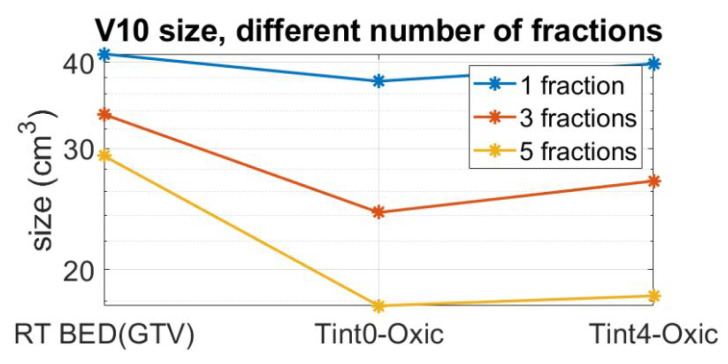
$V_{10}$ for compensated plans so that even with fractionation, we can still achieve BED equal to 37.5 Gy for 99.4% of the voxels in the target.

As a result of fractionation, the BED inevitably decreases. In order to achieve the same therapeutic outcome for the target, specified by the BED = 37.5 Gy, the radiation dose needs to be magnified by an appropriate scale factor, which increases with an increased number of fractions. To assess the effect of fractionation as well as to quantify the impact of the combined plan outside GTV, the $V_{10}$ values for three fraction schemes and two RT + HT time intervals are reported in Figure 6. Observe that the reported fractionated plans are compensated by their respective scaling factors to give the same BED in the target as a single fraction scheme. The combined administration of HT with RT reduces the $V_{10}$ volume, and this reduction is pronounced with an increased number of fractions. In a five-fraction scheme, the $V_{10}$ volume is reduced from 30 cm${}^{3}$ to approximately 17 cm${}^{3}$ for both time intervals of 0 and 4 h.

**Figure 7:**
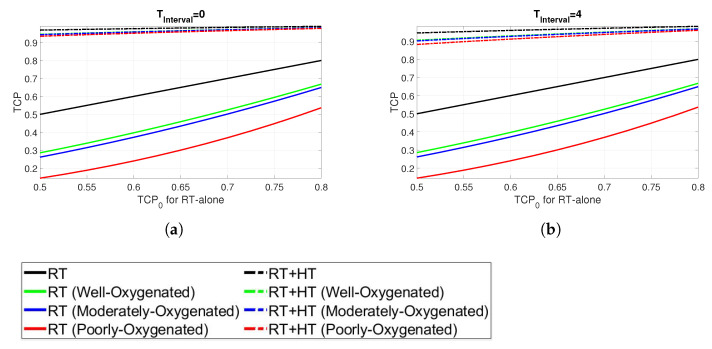
TCP at time intervals (**a**) 0 h and (**b**) 4 h.

Finally, the results in terms of TCP are shown in Figure 7. The TCP for the combined HT and RT is shown in comparison with the TCP for RT alone in the range of [0.5–0.8] and denoted by $TCP_{0}$. In particular, $TCP_{0}$ is based on the assumption that the radiosensitivity of all the cells in the tumour is described by the generic parameters derived in oxic conditions, as given in Table 2. The TCP is calculated for the RT + HT treatment through Equation (12) assuming an average clonogenic cell density determined from $TCP_{0}$. Again, the results are presented for different oxygenation conditions and for time intervals of 0 and 4, respectively (Figure 7a,b). The RT + HT combined plans, represented by dashed lines, exhibit substantially higher TCP than the corresponding RT alone curves. Furthermore, the enhanced effect of combined treatment is more pronounced for cells in hypoxic conditions. The impact of the time interval between the RT and HT is not prominent, as TCP values of the combined plans for both time intervals exhibit the TCP above 0.9 in all cases.

## 4. Discussion

In this methodological study, we implemented the LQ model adapted for thermoradiotherapy [33] and determined clinically relevant parameters for assessing the combined effect of SRS and mild hyperthermia. We further extended the original model by oxygen modification factor [49]. That way, we could account for the well-known ability of hyperthermia to enhance tumour radiosensitivity.

Focused intracranial heating is challenging due to strict constrains on maximum temperatures of 42 °C [57,58]. The patient specific applicator design along with the multi-frequency treatment planning resulted in adequate tumour coverage represented by $T_{90}=39.0\;{}^{\circ}C$, $T_{50}=39.8\;{}^{\circ}C$ and $T_{10}=40.4\;{}^{\circ}C$. The size of the target, 34 cc, is considered to be a large volume for a Gamma Knife treatment, and therefore, many shots were required to reach high-quality metrics, such as coverage, selectivity, and gradient index. The new inverse planner for Gamma Knife treatments, Leksell Gamma Knife^®^ Lightning, leads to plans with more shots than manual planning. Lightning often generates plans with more than one shot in a given isocenter to enhance the dose sculpting properties, which is reflected in Table 1. Note that although many shots are used, the beam-on-time for this large target is not particularly long.

The original, single fraction Gamma Knife treatment plan specified by the BED of 37.5 Gy, corresponding to a prescription dose of 15 Gy, gives a $V_{10}$ of 43 cm${}^{3}$. Since a large $V_{10}$ volume has been found to correlate to adverse cognitive effects, see, for example, [59], we used a five-fraction scheme in the analysis of the boosting effect of thermal therapy. Furthermore, we could show that the combined administration of HT with RT can halve the $V_{10}$ volume in a five-fraction scheme while maintaining the BED in the target. In certain cases, such as in multi-organ metastasis treatments, the size of the low dose volume $V_{10}$ is a highly relevant parameter for treatment planning. In these cases, organs at risk (OAR) are often delineated and inspected specifically for the delivered dose levels.

A significant improvement of the examined parameters, that is, the BED and TCP, was achieved for the combined treatment, indicating a beneficial effect of elevated tumour temperatures. However, it must be stated that the calculated quantitative gains might be affected by the uncertainties in reported values of LQ parameters. Uncertainties in the values of α, β, and α/β, categorized typically by tumour sites, are generally large. In the absence of more specific values, we applied parameters for a generic, early reacting tumour [47]. This is a conservative approach which assumes that the cells are rapidly proliferating and hence have a higher sensitivity to fractionation. A similar strategy was used in previous modelling studies that assessed the trend of the investigated parameters related to the tumour response such as BED and TCP instead of actual quantitative estimates of these parameters [60]. The $\alpha {}_{41}/\alpha {}_{37}$, $\beta {}_{41}/\beta {}_{37}$ were kept the same for both tumour and healthy tissues, as those parameters are unknown for healthy tissue cells. Recently, the thermal dependence of cervical tumour cell lines SiHa and HeLa was experimentally determined by in vitro studies [33,34]. A difference between in vitro and in vivo for some parameters, α/β ratio in particular, is anticipated. Since the difference between the radiobiological parameters for brain tumour cell lines might be even larger than that of cervical cell lines, the results achieved in this study are illustrative.

Furthermore, these results can still be considered as a conservative assessment of the enhanced effect associated with hyperthermia. The LQ model and its extensions do not consider important features of hyperthermia, such as modulation of immunologic responses or changes in tumour microenvironment. In particular, an increased blood flow is expected to enhance the killing effect in hypoxic tumours.

The impact of the time interval between radiotherapy and hyperthermia delivery, in terms of both TCP and BED, appears less important than expected from reviews of radio-biological studies [61,62]. Nevertheless, the enhanced effect appears consistent for different hypoxic conditions. In the context of technological requirements for sequential administration, this is a positive observation that strengthens the combined therapy’s feasibility.

The main impact of this study, apart from demonstrating the potential application of thermoradiotherapy in brain tumour management, is the guidance for evaluation and quantification of the common biological effect of both therapies. Kok et al. [63] suggested the use of equivalent radiation dose (EQD) instead of the cell survival model. We propose to direct the analysis towards the BED, which provides a more straightforward clinical insight and is often used for clinical decisions [64]. Furthermore, for the SRS, we recommend applying the second-order equation to calculate the EQD instead of the first-order solution proposed by [33,65]. Given that dose distributions of SRS are typically more inhomogeneous than that of EBRT, our approach avoids any approximation and thus yields more precise results.

In this study, the combined treatment is demonstrated on a paediatric tumour. However, the use of the Gamma Knife stereotactic radiosurgery (SRS) is not limited by the age of the patients. Rather, the Gamma Knife SRS is widely used in the treatment of both children and adults, primarily when the number of tumours is limited and their volumes small (ideally < 2 cubic centimetres). In pediatric patients, it is important to minimize the dose to the surrounding healthy tissue and thus to reduce the risk of late complications. In adult patients, the Gamma Knife SRS, potentially in combination with hyperthermia, can be particularly useful in the treatment of tumour residues, meningiomas, or metastases.

## 5. Conclusions

This study is the first to propose a methodological concept that evaluates a treatment plan combining stereotactic radiosurgery with microwave hyperthermia. Radiosensitisation has been modelled using an extended version of the LQ model with temperature-dependent radiosensitivity parameters and an oxygen modification factor. The results presented in terms of clinically relevant parameters, BED, $V_{10}$ and TCP, indicate that the focused intracranial heating can be used either to boost the dose to the GTV area or to minimize the dose given to healthy tissues while maintaining the therapeutic effect described by BED. The estimated tumour control can be significantly improved by adjuvant hyperthermia. However, the results should not be seen in terms of absolute gain as they are achieved for this particular and generic case of radiosensitivity parameters.

This study is also the first to advocate a combination of stereotactic radiosurgery with focused heating. It motivates the future development of hyperthermia systems for brain cancer treatment to facilitate clinical trials and validate the effects of the combined treatment. Moreover, the methodological concept proposed here is independent of the form of RT or HT delivery. Therefore, a similar assessment can be performed for virtually any treatment; both EBRT and SRS plans can be applied, as well as heating by other focused delivery modalities, such as ultrasound.

## Figures and Tables

**Table 1:** Metrics for the medulloblastoma plan.

Prescription Dose	Coverage	Selectivity	Gradient Index	Beam-on Time (min) @ 3 Gy/min	Number of Shots
15 Gy	0.994	0.850	2.63	54.8	110

**Table 2:** Parameters for the model in Equation (4).

Parameters	Tumour	Healthy Tissue
$\alpha {}_{37}$ (Gy${}^{-1}$)	0.35	0.35
$\alpha {}_{37}/\beta {}_{37}$ (Gy)	10	3
$\alpha {}_{41}/\alpha {}_{37}$	2.36	2.36
$\beta {}_{41}/\beta {}_{37}$	0.53	0.53
μ (h${}^{-1}$)	0.047	1
ΔS (cal/°C/mol)	423.14	423.14
ΔH (cal/mol)	157,312.3	157,312.3

## Data Availability

Data available on request due to restrictions eg privacy or ethical. The data presented in this study are stored on secure servers and available on request from the corresponding author.

## Abbreviations

The following abbreviations are used in this manuscript:

HTP	Hyperthermia Treatment Planning
PLD	Power Loss Density
SAR	Specific Absorption Rate
GTV	Gross tumour Volume
DVH	Dose Volume Histogram
EQD	EQuivalent radiation Dose
BED	Biologically Effective Dose
TCP	tumour Control Probability

## References

[1] Rosychuk R.J., Witol A., Wilson B., Stobart K. (2012). Central nervous system (CNS) tumor trends in children in a western Canadian province: A population-based 22-year retrospective study. J. Neurol..

[2] Prigorowsky M. (2009). Cancerfondsrapporten 2009.

[3] Han J., Kwon S., Won S., Shin Y., Ko J., Lyu C. (2009). Comprehensive clinical follow-up of late effects in childhood cancer survivors shows the need for early and well-timed intervention. Ann. Oncol..

[4] Makale M.T., McDonald C.R., Hattangadi-Gluth J., Kesari S. (2017). Brain irradiation and long-term cognitive disability: Current concepts. Nat. Rev. Neurol..

[5] Overgaard J., Bentzen S., Gonzalez D.G., Hulshof M., Arcangeli G., Dahl O., Mella O. (1995). Randomised trial of hyperthermia as adjuvant to radiotherapy for recurrent or metastatic malignant melanoma. Lancet.

[6] van der Zee J., González D., van Rhoon G.C., van Dijk J.D., van Putten W.L., Hart A.A. (2000). Comparison of radiotherapy alone with radiotherapy plus hyperthermia in locally advanced pelvic tumours: A prospective, randomised, multicentre trial. Lancet.

[7] Datta N.R., Rogers S., Ordóñez S.G., Puric E., Bodis S. (2016). Hyperthermia and radiotherapy in the management of head and neck cancers: A systematic review and meta-analysis. Int. J. Hyperth..

[8] Datta N.R., Rogers S., Klingbiel D., Gómez S., Puric E., Bodis S. (2016). Hyperthermia and radiotherapy with or without chemotherapy in locally advanced cervical cancer: A systematic review with conventional and network meta-analyses. Int. J. Hyperth..

[9] Datta N.R., Puric E., Klingbiel D., Gomez S., Bodis S. (2016). Hyperthermia and radiation therapy in locoregional recurrent breast cancers: A systematic review and meta-analysis. Int. J. Radiat. Oncol. Biol. Phys..

[10] Issels R.D., Lindner L.H., Verweij J., Wust P., Reichardt P., Schem B.C., Abdel-Rahman S., Daugaard S., Salat C., Wendtner C.M. (2010). Neo-adjuvant chemotherapy alone or with regional hyperthermia for localised high-risk soft-tissue sarcoma: A randomised phase 3 multicentre study. Lancet Oncol..

[11] Issels R.D., Lindner L.H., Verweij J., Wessalowski R., Reichardt P., Wust P., Ghadjar P., Hohenberger P., Angele M., Salat C. (2018). Effect of neoadjuvant chemotherapy plus regional hyperthermia on long-term outcomes among patients with localized high-risk soft tissue sarcoma: The EORTC 62961-ESHO 95 randomized clinical trial. JAMA Oncol..

[12] Peeken J.C., Vaupel P., Combs S.E. (2017). Integrating hyperthermia into modern radiation oncology: What evidence is necessary?. Front. Oncol..

[13] Ott O.J. (2010). Hyperthermia in Oncology: Principles and Therapeutic Outlook.

[14] Wessalowski R., Schneider D.T., Mils O., Friemann V., Kyrillopoulou O., Schaper J., Matuschek C., Rothe K., Leuschner I., Willers R. (2013). Regional deep hyperthermia for salvage treatment of children and adolescents with refractory or recurrent non-testicular malignant germ-cell tumours: An open-label, non-randomised, single-institution, phase 2 study. Lancet Oncol..

[15] Hulshof M., Raaymakers B., Lagendijk J., Koot R., Crezee H., Stalpers L., Gonzalez Gonzalez D. (2004). A feasibility study of interstitial hyperthermia plus external beam radiotherapy in glioblastoma multiforme using the multi electrode current source (MECS) system. Int. J. Hyperth..

[16] Salcman M., Samaras G.M. (1983). Interstitial microwave hyperthermia for brain tumors. J. Neuro-Oncol..

[17] Winter A., Laing J., Paglione R., Sterzer F. (1985). Microwave hyperthermia for brain tumors. Neurosurgery.

[18] Sneed P.K., Stauffer P.R., McDermott M.W., Diederich C.J., Lamborn K.R., Prados M.D., Chang S., Weaver K.A., Spry L., Malec M.K. (1998). Survival benefit of hyperthermia in a prospective randomized trial of brachytherapy boost ± hyperthermia for glioblastoma multiforme. Int. J. Radiat. Oncol. Biol. Phys..

[19] Shirvalilou S., Khoei S., Esfahani A.J., Kamali M., Shirvaliloo M., Sheervalilou R., Mirzaghavami P. (2021). Magnetic Hyperthermia as an adjuvant cancer therapy in combination with radiotherapy versus radiotherapy alone for recurrent/progressive glioblastoma: A systematic review. J. Neuro-Oncol..

[20] Jordan A., Maier-Hauff K. (2007). Magnetic nanoparticles for intracranial thermotherapy. J. Nanosci. Nanotechnol..

[21] Wankhede M., Bouras A., Kaluzova M., Hadjipanayis C.G. (2012). Magnetic nanoparticles: An emerging technology for malignant brain tumor imaging and therapy. Expert Rev. Clin. Pharmacol..

[22] Giammalva G.R., Gagliardo C., Marrone S., Paolini F., Gerardi R.M., Umana G.E., Yağmurlu K., Chaurasia B., Scalia G., Midiri F. (2021). Focused Ultrasound in Neuroscience. State of the Art and Future Perspectives. Brain Sci..

[23] Turner P., Tumeh A., Schaefermeyer T. (1989). BSD-2000 approach for deep local and regional hyperthermia: Physics and technology. Strahlenther. Onkol..

[24] Paulides M., Bakker J., Neufeld E., Zee J.v.d., Jansen P., Levendag P., Van Rhoon G. (2007). The HYPERcollar: A novel applicator for hyperthermia in the head and neck. Int. J. Hyperth..

[25] Takook P., Persson M., Trefná H.D. (2018). Performance evaluation of hyperthermia applicators to heat deep-seated brain tumors. IEEE J. Electromagn. RF Microwaves Med. Biol..

[26] Rodrigues D., Ellsworth J., Turner P. (2021). Feasibility of heating brain tumors using a 915 MHz annular phased array. IEEE Antennas Wirel. Propag. Lett..

[27] Winter L., Özerdem C., Hoffmann W., Santoro D., Müller A., Waiczies H., Seemann R., Graessl A., Wust P., Niendorf T. (2013). Design and evaluation of a hybrid radiofrequency applicator for magnetic resonance imaging and RF induced hyperthermia: Electromagnetic field simulations up to 14.0 Tesla and proof-of-concept at 7.0 Tesla. PLoS ONE.

[28] Zanoli M., Trefna H.D. (2021). Iterative time-reversal for multi-frequency hyperthermia. Phys. Med. Biol..

[29] Oberacker E., Kuehne A., Oezerdem C., Nadobny J., Weihrauch M., Beck M., Zschaeck S., Diesch C., Eigentler T.W., Waiczies H. (2020). Radiofrequency applicator concepts for thermal magnetic resonance of brain tumors at 297 MHz (7.0 Tesla). Int. J. Hyperth..

[30] Trefná H.D., Martinsson B., Petersson T., Renström N., Torstensson M., Ravanis J., Kok P., Persson M. Multifrequency approach in hyperthermia treatment planning: Impact of frequency on SAR distribution in head and neck. Proceedings of the 2017 11th European Conference on Antennas and Propagation (EUCAP).

[31] Schooneveldt G., Trefná H.D., Persson M., De Reijke T.M., Blomgren K., Kok H.P., Crezee H. (2019). Hyperthermia treatment planning including convective flow in cerebrospinal fluid for brain tumour hyperthermia treatment using a novel dedicated paediatric brain applicator. Cancers.

[32] Pollock B.E. (2019). Complications After Stereotactic Radiosurgery. Complications in Neurosurgery.

[33] Van Leeuwen C., Oei A., Ten Cate R., Franken N., Bel A., Stalpers L., Crezee J., Kok H. (2018). Measurement and analysis of the impact of time-interval, temperature and radiation dose on tumour cell survival and its application in thermoradiotherapy plan evaluation. Int. J. Hyperth..

[34] van Leeuwen C., Crezee J., Oei A., Franken N., Stalpers L., Bel A., Kok H. (2018). The effect of time interval between radiotherapy and hyperthermia on planned equivalent radiation dose. Int. J. Hyperth..

[35] Alper T., Howard-Flanders P. (1956). Role of oxygen in modifying the radiosensitivity of *E. coli* B. Nature.

[36] (2016). Tissue Properties Database. V3.1. http://itis.swiss/virtual-population/tissue-properties/downloads-v3-1/.

[37] Rossmann C., Haemmerich D. (2014). Review of temperature dependence of thermal properties, dielectric properties, and perfusion of biological tissues at hyperthermic and ablation temperatures. Crit. Rev. Biomed. Eng..

[38] Lang J., Erdmann B., Seebass M. (1999). Impact of nonlinear heat transfer on temperature control in regional hyperthermia. IEEE Trans. Biomed. Eng..

[39] Takook P., Persson M., Gellermann J., Trefná H.D. (2017). Compact self-grounded Bow-Tie antenna design for an UWB phased-array hyperthermia applicator. Int. J. Hyperth..

[40] Canters R., Franckena M., van der Zee J., Van Rhoon G. (2010). Optimizing deep hyperthermia treatments: Are locations of patient pain complaints correlated with modelled SAR peak locations?. Phys. Med. Biol..

[41] Zanoli M., Trefná H.D. Optimization of microwave hyperthermia array applicators using field interpolation. Proceedings of the 2019 IEEE International Symposium on Antennas and Propagation and USNC-URSI Radio Science Meeting.

[42] Kennedy J., Eberhart R. Particle swarm optimization. Proceedings of the ICNN’95-International Conference on Neural Networks.

[43] Yarmolenko P.S., Moon E.J., Landon C., Manzoor A., Hochman D.W., Viglianti B.L., Dewhirst M.W. (2011). Thresholds for thermal damage to normal tissues: An update. Int. J. Hyperth..

[44] Lindquist C., Paddick I. (2007). The Leksell Gamma Knife Perfexion and comparisons with its predecessors. Oper. Neurosurg..

[45] Torrens M., Chung C., Chung H.T., Hanssens P., Jaffray D., Kemeny A., Larson D., Levivier M., Lindquist C., Lippitz B. (2014). Standardization of terminology in stereotactic radiosurgery: Report from the Standardization Committee of the International Leksell Gamma Knife Society: Special topic. J. Neurosurg..

[46] Dewey W., Hopwood L., Sapareto S., Gerweck L. (1977). Cellular responses to combinations of hyperthermia and radiation. Radiology.

[47] Fowler J.F. (2009). Sensitivity analysis of parameters in linear-quadratic radiobiologic modeling. Int. J. Radiat. Oncol. Biol. Phys..

[48] Overgaard J. (1980). Simultaneous and sequential hyperthermia and radiation treatment of an experimental tumor and its surrounding normal tissue in vivo. Int. J. Radiat. Oncol. Biol. Phys..

[49] Toma-Dasu I., Dasu A. (2013). Modelling tumour oxygenation, reoxygenation and implications on treatment outcome. Comput. Math. Methods Med..

[50] Alper T. (1979). Cellular Radiobiology.

[51] Hall E.J., Giaccia A.J. (2006). Radiobiology for the Radiologist.

[52] Vaupel P., Multhoff G. (2017). Accomplices of the hypoxic tumor microenvironment compromising antitumor immunity: Adenosine, lactate, acidosis, vascular endothelial growth factor, potassium ions, and phosphatidylserine. Front. Immunol..

[53] Daşu A., Toma-Daşu I. (2008). Treatment modelling: The influence of micro-environmental conditions. Acta Oncol..

[54] Brahme A. (1999). Optimized radiation therapy based on radiobiological objectives. Seminars in Radiation Oncology.

[55] Dewey W.C. (1994). Arrhenius relationships from the molecule and cell to the clinic. Int. J. Hyperth..

[56] van Rhoon G.C. (2016). Is CEM43 still a relevant thermal dose parameter for hyperthermia treatment monitoring?. Int. J. Hyperth..

[57] van der Zee J., Vujaskovic Z., Kondo M., Sugahara T. (2008). The Kadota fund international forum 2004–Clinical group consensus. Int. J. Hyperth..

[58] Haveman J., Sminia P., Wondergem J., van der Zee J., Hulshof M. (2005). Effects of hyperthermia on the central nervous system: What was learnt from animal studies?. Int. J. Hyperth..

[59] Minniti G., Clarke E., Lanzetta G., Osti M.F., Trasimeni G., Bozzao A., Romano A., Enrici R.M. (2011). Stereotactic radiosurgery for brain metastases: Analysis of outcome and risk of brain radionecrosis. Radiat. Oncol..

[60] Lindblom E., Toma-Dasu I., Dasu A. (2018). Accounting for two forms of hypoxia for predicting tumour control probability in radiotherapy: An in silico study. Oxygen Transport to Tissue XL.

[61] Horsman M., Overgaard J. (2007). Hyperthermia: A potent enhancer of radiotherapy. Clin. Oncol..

[62] Oei A., Kok H., Oei S., Horsman M., Stalpers L., Franken N., Crezee J. (2020). Molecular and biological rationale of hyperthermia as radio-and chemosensitizer. Adv. Drug Deliv. Rev..

[63] Kok H.P., Crezee J., Franken N.A., Stalpers L.J., Barendsen G.W., Bel A. (2014). Quantifying the combined effect of radiation therapy and hyperthermia in terms of equivalent dose distributions. Int. J. Radiat. Oncol. Biol. Phys..

[64] Fowler J.F. (2010). 21 years of biologically effective dose. Br. J. Radiol..

[65] Van Leeuwen C., Crezee J., Oei A., Franken N., Stalpers L., Bel A., Kok H. (2017). 3D radiobiological evaluation of combined radiotherapy and hyperthermia treatments. Int. J. Hyperth..

